# Preventive Effect of Butyrate in Colon Cancer Cell Metabolism

**DOI:** 10.3390/ijms27083696

**Published:** 2026-04-21

**Authors:** Telmo José Gonçalves, Ana Margarida Abrantes, Ana Salomé Pires, Ana Cristina Gonçalves, Ludgero Canário Tavares, João Casalta-Lopes, Ana Bela Sarmento-Ribeiro, Rui A. Carvalho, Maria Filomena Botelho

**Affiliations:** 1 Univ Coimbra, Coimbra Institute for Clinical and Biomedical Research (iCBR) area of Environment Genetics and Oncobiology (CIMAGO), Biophysics Institute of Faculty of Medicine, 3000-548 Coimbra, Portugal; uc2008115837@student.bioq.uc.pt (T.J.G.); mabrantes@fmed.uc.pt (A.M.A.); mfbotelho@fmed.uc.pt (M.F.B.); 2 Univ Coimbra, Faculty of Pharmacy, 3000-548 Coimbra, Portugal; 3 Univ Coimbra, Center for Innovative Biomedicine and Biotechnology (CIBB), 3000-548 Coimbra, Portugal; acgoncalves@fmed.uc.pt (A.C.G.); absarmento@fmed.uc.pt (A.B.S.-R.); 4 Clinical Academic Center of Coimbra (CACC), 3004-561 Coimbra, Portugal; 5 Univ Coimbra, Coimbra Institute for Clinical and Biomedical Research (iCBR) area of Environment Genetics and Oncobiology (CIMAGO), Laboratory of Oncobiology and Hematology and University Clinics of Hematology and Oncology, Faculty of Medicine, 3000-548 Coimbra, Portugal; 6 Univ Coimbra, CNC-Center for Neuroscience and Cell Biology, 3004-504 Coimbra, Portugal; 7 Vasco da Gama Research Center (CIVG), University School Vasco da Gama (EUVG), 3020-210 Coimbra, Portugal; 8 Coimbra Health School, Polytechnic University of Coimbra, 3045-093 Coimbra, Portugal; joao.casalta@estesc.ipc.pt; 9 Department of Radiotherapy, Unidade Local de Saúde de São João, 4200-319 Porto, Portugal; 10 Life and Health Sciences Research Institute (ICVS), School of Medicine, University of Minho, 4710-057 Braga, Portugal; 11 Univ Coimbra, Faculty of Pharmacy, REQUIMTE/LAQV, Group of Pharmaceutical Technology, 3000-548 Coimbra, Portugal; carvalho@uc.pt; 12 Univ Coimbra, Faculty of Sciences and Technology, Department of Life Sciences, 3000-456 Coimbra, Portugal

**Keywords:** butyrate, colorectal cancer, Warburg effect, glucose transporters, metabolic reprogramming

## Abstract

Butyrate, a short-chain fatty acid produced by the fermentation of soluble dietary fiber by gut bacteria, also functions as a histone deacetylase inhibitor known to induce apoptosis and promote differentiation in colon tumor cells. During tumorigenesis, cancer cells undergo metabolic reprogramming to meet energetic and biosynthetic demands, increasing glycolytic metabolism and reducing oxidative metabolism—a phenomenon known as the Warburg effect. This study aimed to evaluate the impact of butyrate on the aggressiveness-related metabolic phenotype of three colon cancer cell lines (LS1034, C2BBe1, and WiDr). Butyrate’s effects were assessed through fluorine-18 fluorodeoxyglucose ([^18^F]FDG) uptake, flow cytometry analysis of cytoplasmic and membrane expression of glucose transporters (GLUT1, GLUT3, GLUT5, and GLUT12), lactate production, and analysis of Krebs cycle turnover and glycolysis–Krebs cycle coupling using nuclear magnetic resonance isotopomer profiling. [^18^F]FDG uptake decreased in C2BBe1 and WiDr cells, whereas an opposite response was observed in LS1034 cells, which also exhibited reduced GLUT5 expression. These uptake patterns were consistent with lactate production measurements, and an enhancement of oxidative metabolism was detected in C2BBe1 and WiDr cells. Although butyrate was consumed by all three cell lines, its metabolic handling appeared to differ in LS1034 cells, possibly reflecting cytotoxic stress and/or distinct metabolic regulation mechanisms. Overall, these findings indicate that butyrate exerts cell-line-dependent metabolic effects in colorectal cancer cells. In C2BBe1 and WiDr cells, butyrate exposure was broadly consistent with the attenuation of glycolytic/Warburg-associated features, whereas LS1034 cells displayed a divergent response and were interpreted separately. These data support further investigation of butyrate as a modulator of colorectal cancer cell metabolism, while highlighting the heterogeneity of metabolic responses across tumor models.

## 1. Introduction

Colorectal cancer (CRC) remains a major global health concern, ranking as the third most common cancer and the second leading cause of cancer-related mortality worldwide, highlighting the need for effective prevention and treatment strategies [1,2]. Emerging evidence indicates that butyrate, a short-chain fatty acid (SCFA) produced by microbial fermentation of soluble dietary fiber, is a key tumor-suppressive metabolite. Whereas normal differentiated colonocytes rely on butyrate to supply 60–70% of their energy through mitochondrial β-oxidation (thereby maintaining epithelial homeostasis), CRC cells exhibit the Warburg effect, characterized by a metabolic shift toward aerobic glycolysis and increased glucose consumption to sustain high proliferative demands [2,3]. This metabolic divergence underlies the “butyrate paradox”; in cancer cells, butyrate is less efficiently oxidized and therefore accumulates in the nucleus, where it functions as a histone deacetylase inhibitor (HDACi) that modulates gene expression, induces apoptosis, and suppresses proliferation [4,5,6].

During carcinogenesis, tumor cells develop metabolic alterations that support the energetic and biosynthetic requirements of exacerbated proliferation. As a result, they intensify glycolytic flux while reducing the oxidative metabolism (*Warburg* effect) [6]. This metabolic reprogramming leads to increased lactate production, enhanced glucose uptake, upregulation of glucose transporters (GLUTs), and reduced acetyl-CoA availability due to impaired pyruvate entry into the mitochondria [7]. A more pronounced *Warburg* phenotype is typically associated with higher proliferative capacity and greater tumor aggressiveness [8,9]. Consequently, CRC cells rely predominantly on glucose rather than butyrate as their main energy source. However, several studies suggest that butyrate can compete with glucose metabolism and counteract some of the metabolic alterations acquired during tumor progression [10,11].

Understanding how butyrate interferes with this aggressive metabolic phenotype—particularly the Warburg effect—remains an important research goal. Recent mechanistic studies have shed light on butyrate’s anti-Warburg activity [12,13]. Studies in the highly glycolytic HCT116 CRC cell line have demonstrated that butyrate effectively suppresses the Warburg effect and induces a metabolic shift from an energetically active glycolytic state toward a more oxidative and quiescent phenotype. This reprogramming mechanism involves direct targeting of a key glycolytic enzyme, the M2 isoform of pyruvate kinase (PKM2) [13]. Butyrate binds to and activates PKM2, promoting its dephosphorylation and tetramerization, which leads to a significant accumulation of pyruvate, decreased upstream glycolytic intermediates, and inhibition of anabolic processes essential for rapid tumor proliferation, such as nucleotide synthesis. As a result, ATP production becomes less dependent on glycolysis and increasingly reliant on oxidative phosphorylation (OXPHOS) [12,13].

This study was designed to investigate the interplay between butyrate-induced oxidative metabolism and the glycolytic phenotype characteristics of three human colon cancer cell lines. To this end, we evaluated the effects of butyrate on [^18^F]FDG uptake, GLUT expression, lactate production, glycolytic activity, glycolysis–Krebs cycle coupling, and Krebs cycle flux.

## 2. Results

### 2.1. [^18^F]FDG Uptake Studies

**Figure 1:**
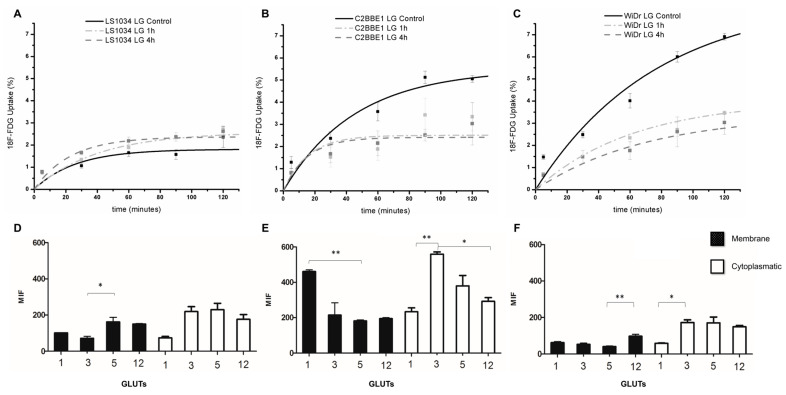
[^18^F]FDG uptake studies (**A**–**C**). Evaluation of [^18^F]FDG uptake by LS1034 (**A**), C2BBe1 (**B**), and WiDr (**C**) cells after incubation in the absence (control) or presence of butyrate for 1 and 4 h. Data represent the mean ± SD of four independent experiments performed in duplicate. Basal membrane and cytoplasmic expression levels (time 0, untreated) of GLUT1, GLUT3, GLUT5, and GLUT12 in LS1034 (**D**), C2BBe1 (**E**), and WiDr (**F**) colon cancer cell lines. Results are presented as mean intensity fluorescence (MIF) and represent the mean ± SD of three independent experiments. Statistical significance: * *p* < 0.05; ** *p* < 0.01.

In the LS1034 cell line, 4 h of incubation with butyrate induced an increase in [^18^F]FDG uptake compared with control cells (Figure 1A), from 1.83 ± 0.15% to 2.54 ± 0.22% of maximum uptake (Table 1).

In contrast, in the C2BBe1 and WiDr cell lines, butyrate induced a decrease in [^18^F]FDG uptake (Figure 1B,C). In the C2BBe1 cell line, exposure to butyrate for 1 and 4 h decreased maximum uptake from 7.22 ± 0.99% (control) to 3.71 ± 1.22% and 2.66 ± 0.42%, respectively (Table 1). In WiDr cells, maximum uptake decreased from 11.82 ± 5.00% to 4.11 ± 0.29% after 1 h of butyrate exposure and to 2.95 ± 0.34% after 4 h.

### 2.2. Expression of GLUT1, GLUT3, GLUT5, and GLUT12

In the first stage, basal membrane and cytoplasmic levels of the four GLUTs (Figure 1D–F) were determined in the three cell lines in the absence of butyrate. In the LS1034 cell line, all four GLUT isoforms were expressed at membrane and cytoplasmic levels. GLUT1 and GLUT3 were the least expressed membrane transporters (*p* = 0.020), whereas GLUT5 and GLUT12 showed the highest membrane expression, being approximately 1.5-fold more abundant than GLUT1.

In C2BBe1 cells (Figure 1E), GLUT3 was the most abundantly expressed cytoplasmic transporter, being 2.4-fold and 1.9-fold higher than GLUT1 (*p* = 0.001) and GLUT12 (*p* = 0.024), respectively. Conversely, GLUT1 displayed the highest membrane expression.

In WiDr cells (Figure 1F), GLUT1 was the least expressed cytoplasmic transporter compared with GLUT3 (*p* = 0.022). Regarding membrane expression, GLUT12 was the most abundant carrier, 1.6-fold more than the second most expressed transporter, GLUT1, and 2.4-fold more than the least expressed transporter, GLUT5 (*p* = 0.001).

In the second stage, the effects of 1 and 24 h of butyrate exposure on membrane and cytoplasmic GLUT expression were evaluated.

**Figure 2:**
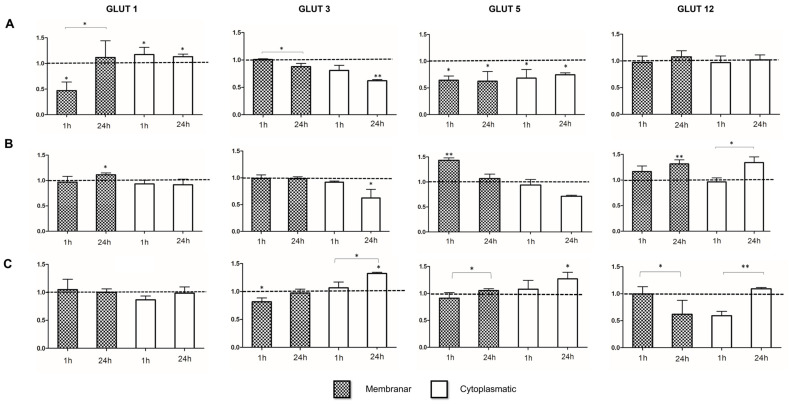
GLUT expression in LS1034 (**A**), C2BBe1 (**B**), and WiDr (**C**) cells after 1 or 24 h of butyrate exposure. Values represent mean intensity fluorescence normalized to untreated controls and are expressed as mean ± SD of four independent experiments. Statistical significance: * *p* < 0.05; ** *p* < 0.01.

In LS1034 cells (Figure 2A), after 1 h of butyrate exposure, membrane expression of GLUT1 decreased to 0.47 ± 0.17 (*p* = 0.020), whereas cytoplasmic expression increased to 1.13 ± 0.05 (*p* = 0.015). Both membrane and cytoplasmic GLUT5 expression decreased to 0.64 ± 0.08 (*p* = 0.040) and 0.68 ± 0.16 (*p* = 0.031), respectively. After 24 h of butyrate exposure, cytoplasmic GLUT1 expression increased to 1.13 ± 0.05 (*p* = 0.015), cytoplasmic GLUT3 expression decreased to 0.62 ± 0.02 (*p* = 0.002), and membrane and cytoplasmic GLUT5 expression decreased to 0.63 ± 0.18 (*p* = 0.014) and 0.75 ± 0.04 (*p* = 0.031), respectively. When comparing 1 h and 24 h timepoints, membrane GLUT1 expression increased (*p* = 0.032) and membrane GLUT3 expression decreased (*p* = 0.011).

In C2BBe1 cells (Figure 2B), butyrate increased expression of GLUT1, GLUT5, and GLUT12 under several conditions. After 1 h of exposure, membrane expression of GLUT5 increased to 1.43 ± 0.05 in (*p* = 0.006). After 24 h, membrane expression of GLUT1 and GLUT12 increased to 1.11 ± 0.03 (*p* = 0.042) and to 1.31 ± 0.08 (*p* = 0.03), respectively, while GLUT3 cytoplasmic expression decreased (*p* = 0.02).

In WiDr cells (Figure 2C), 1 h of butyrate exposure reduced cytoplasmic GLUT1 expression (*p* = 0.025) and membrane GLUT3 expression (*p* = 0.048). In contrast, 24 h exposure increased cytoplasmic GLUT3 and GLUT5 expression to 1.32 ± 0.02 (*p* = 0.021) and to 1.27 ± 0.12 (*p* = 0.037), respectively. Comparing 1 h and 24 h exposures, cytoplasmatic GLUT3 (*p* = 0.041) and membrane GLUT5 (*p* = 0.041) and cytoplasmic GLUT12 (*p* = 0.041) expression increased after 24 h, whereas membrane GLUT12 expression decreased (*p* = 0.025).

### 2.3. Metabolic Studies by NMR

**Figure 3:**
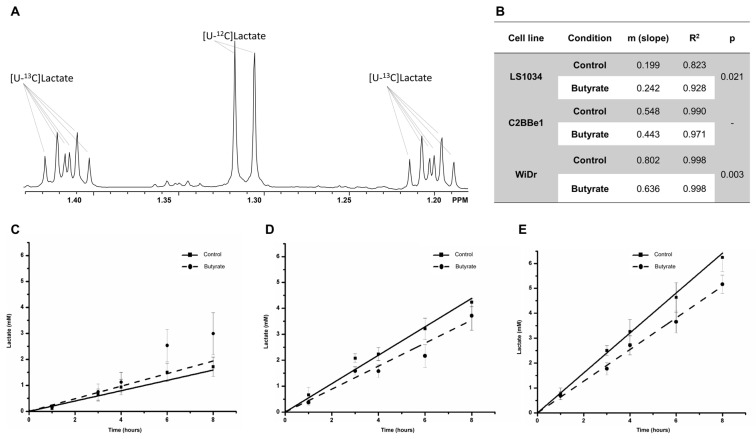
Expansion of the region of the ^1^H NMR spectrum from cell culture medium (**A**) showing the resonances due to the methyl resonance of unenriched lactate ([U-^12^C]lactate) and the two ^13^C satellites of the methyl resonance of uniformly enriched lactate ([U-^13^C]lactate). (**B**) Table with the values of the slopes (m) of the accumulation of [U-^13^C]lactate during 8 h of incubation with or without butyrate for the cell lines LS1034 (**C**), C2BBe1 (**D**), and WiDr (**E**). Values express the mean and standard deviation of four independent experiments. The value m corresponds to the slope of the linear response.

Figure 3 depicts the temporal evolution of [U-^13^C]lactate production in the three cell lines, in the presence or absence of butyrate. A linear response was obtained for all cell lines (Figure 3B). In LS1034 cells, butyrate increased [U-^13^C]lactate production (*p* = 0.021) with greater differences observed at longer incubation times. In C2BBe1 cells, the largest difference occurred at 6 h (1.0 mM), whereas in WiDr cells, the greatest difference was detected at 8 h (1.1 mM). In WiDr cells, the slope was lower under butyrate treatment (m = 0.636) compared with control (m = 0.802; *p* = 0.003). Among the three lines, WiDr control cells showed the highest slope (m = 0.802), indicating greater glycolytic activity and a more intense Warburg phenotype, whereas LS1034 cells exhibited the lowest slope (m = 0.198), consistent with lower glycolytic activity [14].

**Figure 4:**
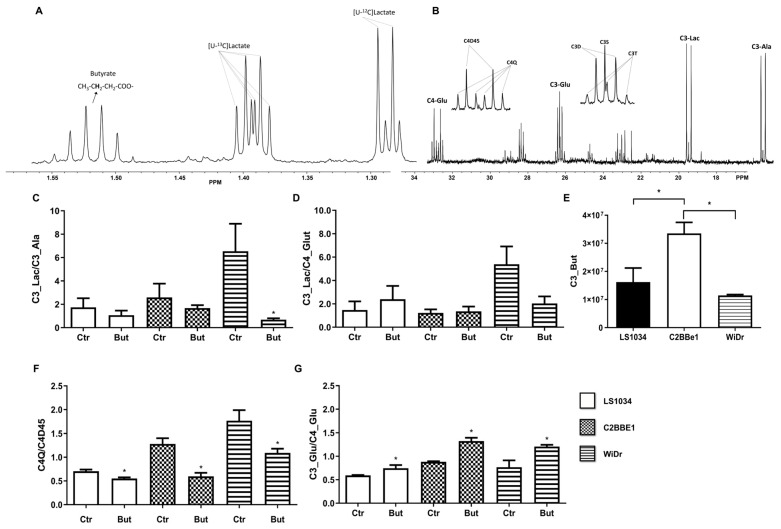
Expansions of the ^1^H (**A**) and the ^13^C (**B**) NMR spectra of an extract from WiDr cells incubated with cell culture media supplemented with [U-^13^C]glucose and natural-abundance butyrate. In each expansion are signaled the resonances of metabolites used in the analysis for deriving metabolic parameters for the three colorectal cancer cell lines in the presence or absence of butyrate, including C3-Glu (carbon 3 of glutamate), C4-Glu (carbon 4 of glutamate), C3-Lac (carbon 3 of lactate), C3-Ala (carbon 3 of alanine), C4Q (quartet of glutamate carbon 4), C4D45 (doublet of glutamate carbon 4 corresponding to labeling at carbons 4 and 5), and C3D, C3S, and C3T (doublet, singlet, and triplet of carbon 3, respectively). (**C**) Ratios between C3-Lac and C3-Ala. (**D**) Ratios of ^13^C enrichment of C3-Lac vs. C4-Glu. (**E**) Quantification of intracellular butyrate. (**F**) Ratios of C4Q and C4D45, used as an indicator of Krebs cycle turnover in the context of the overall isotopomer pattern. (**G**) Ratios of ^13^C enrichment in C3-Glu vs. C4-Glu, denoting influence of anaplerosis in ^13^C incorporation in Krebs cycle intermediates. For butyrate-treated conditions, cells were exposed to butyrate for 24 h. Values express the mean and standard deviation of three independent experiences. Statistical significance with the respective control: * *p* < 0.05.

Butyrate significantly decreased the C3-Lac/C3- Ala ratio in WiDr cells compared with control (*p* = 0.034). The C3-Lac/C4-Glut ratio (Figure 4D) reflects the coupling between glycolysis and Krebs cycle. In LS1034 cells, butyrate increased this ratio, suggesting the decoupling of glycolysis and the Krebs cycle and an enhanced Warburg effect. No significant change was observed in C2BBe1 cells. In WiDr cells, butyrate decreased this ratio, indicating improved coupling.

Figure 4E shows butyrate uptake across the three cell lines. C2BBe1 cells exhibited higher uptake compared with LS1034 (*p* = 0.048) and WiDr (*p* = 0.016) cells.

Butyrate significantly decreased the glutamate C4Q/C4D45 ratio (Figure 4F) in LS1034 (*p* = 0.034), C2BBe1 (*p* = 0.049), and WiDr (*p* = 0.034) cells, suggesting a reduced contribution of 13C-labeled substrates to Krebs cycle turnover under butyrate exposure. However, this parameter should not be interpreted in isolation, since natural-abundance butyrate may also dilute the 13C enrichment of Krebs cycle intermediates. In contrast, the C3-Glu/C4-Glu ratio (Figure 4G) increased in LS1034 (*p* = 0.043), C2BBe1 (*p* = 0.049), and WiDr (*p* = 0.049) cells, suggesting an enhanced anaplerotic flux from [U-^13^C]pyruvate.

## 3. Discussion

During tumorigenesis, cells undergo metabolic reprogramming to sustain rapid proliferation and support the biosynthesis of organelles required for deregulated cell division, a phenomenon known as the Warburg effect. To obtain energy quickly, the tumor cell intensifies the glycolytic pathway, decreases Krebs cycle flux, using it essentially for anaplerosis, and decreases oxidative phosphorylation [7,9]. Increased glycolytic flux leads to higher glucose consumption, requiring GLUT overexpression to enhance glucose uptake beyond that of non-tumor cells [15,16].

In this study, we investigated the effect of butyrate on metabolic changes associated with the Warburg effect in three colorectal cancer cell lines and compared the response across the different models. This should be interpreted considering that the use of cell-line-specific IC_50_ concentrations was intended to normalize biological exposure, but does not allow strict quantitative comparison of butyrate effects across the three models. During metabolic reprogramming induced by tumorigenesis, colon cancer cells increase glucose uptake and glycolytic activity [17,18]. [^18^F]FDG uptake studies allow the evaluation of butyrate-induced changes in [^18^F]FDG uptake and, indirectly, in glucose uptake, since [^18^F]FDG is a glucose analog. In WiDr and C2BBe1 cells, butyrate decreased radiopharmaceutical uptake by more than 50%. Since low-glucose conditions more closely mimic euglycemia, uptake experiments were performed under these conditions. In these two cell lines, the results are consistent with a reduction in glucose analog uptake after butyrate exposure. In contrast, LS1034 cells showed increased [^18^F]FDG uptake after exposure to butyrate, indicating that this model does not follow the same pattern observed in WiDr and C2BBe1. This different behavior is in line with the previous metabolic characterization of these models, where LS1034 showed a distinct metabolic profile when compared with WiDr and C2BBe1 cells [14]. A possible explanation is that, in LS1034 cells, butyrate exposure may reflect not only metabolic adaptation but also a more pronounced cytotoxic/stress response, as supported by previous work from our group [19]. In addition, P-glycoprotein expression/activity may influence intracellular tracer retention in this model [19,20]. Therefore, the FDG uptake pattern observed in LS1034 should be interpreted considering both its distinct metabolic background and the cytotoxic effects of butyrate. To further explore whether changes in [^18^F]FDG uptake were associated with changes in glucose transporter expression, GLUT expression was evaluated before and after exposure to butyrate. GLUT1, GLUT3, GLUT5, and GLUT12 are also referred to be [^18^F]FDG transporters [21].

Evaluating the membrane expression of GLUTs is particularly relevant, since membrane-localized transporters mediate glucose and [^18^F]FDG uptake. In contrast, the role of cytoplasmic GLUT expression remains unclear; it is assumed that these transporters serve as a reserve pool that can be recruited to the membrane when required [22]. It was proven that all three colon cancer cell lines express the four isoforms under study at cytoplasmic and membrane levels (Figure 1), as also proven in the literature [21,23,24]. This carrier was also found to be one of the most expressed at the membrane level in the LS1034 cell line (as well as GLUT-5) and the most expressed in the WiDr cell line.

In general, GLUT expression after butyrate exposure was cell line and incubation time dependent. In the LS1034 cell line, butyrate decreased membrane expression of GLUT-1 and GLUT-5 (one of the most expressed at basal level) although [^18^F]FDG uptake increased, contrary to what would be expected if membrane GLUT abundance was the main determinant of tracer uptake [22]. Therefore, membrane GLUT expression alone does not explain the uptake pattern observed in this cell line. This apparent discrepancy may be related to the distinct metabolic profile of LS1034 cells, previously described by our group, and to the stronger cytotoxic effect of butyrate reported for this model. In addition, altered [^18^F]FDG retention related to P-glycoprotein expression cannot be excluded [22]. Thus, LS1034 should be interpreted separately from the other two cell lines. A different pattern was observed in C2BBe1 cells, in which butyrate promoted an increase in the membrane expression of some GLUT isoforms under study. Considering the decrease in [^18^F]FDG uptake after exposure to butyrate, a decrease in membrane expression of some GLUT isoforms would be expected [25]. One possible explanation is that butyrate interferes with GLUT kinetics [26,27], thereby reducing glucose analog transport independently of transporter abundance. Another possibility is that butyrate induces changes in glycoproteins [28], which may affect transporter function at the cell surface. It may also interfere with the expression of glucose transport facilitators such as sodium–glucose linked transporters, which are overexpressed in several tumor types [7], namely in Caco-2 cells, from which C2BBe1 is a clone (ATCC^®^ CRL2102™). In the WiDr cell line, butyrate decreased membrane expression of GLUT-3 after 1 h of exposure, which agrees with the decrease in [^18^F]FDG uptake. Taken together, these findings suggest that the different experimental approaches captured complementary stages of the cellular response to butyrate. The [^18^F]FDG uptake assay reflects an early functional readout of glucose analog handling, whereas GLUT expression may represent later or parallel regulatory changes that do not necessarily translate directly into tracer uptake. The NMR data adds metabolic context by showing whether those functional changes are associated with subsequent alterations in glycolytic activity and glycolysis–Krebs cycle coupling. In the present study, this overall relationship was more consistent in WiDr and C2BBe1 cells than in LS1034 cells, which again supports the interpretation of LS1034 as a distinct model. Since glucose-6-phosphate isomerase does not recognize [^18^F]FDG-6-phosphate due to its structural differences from glucose, [^18^F]FDG taken up by the cell is not metabolized in the same way as glucose and accumulates in the cytoplasm [21]. For this reason, NMR studies using [U-^13^C]glucose were performed to better characterize metabolic changes involving glucose metabolism. Once [U-^13^C]glucose enters the cell through GLUTs, it is converted into two [U-^13^C]pyruvate molecules through glycolysis, which may then be converted into [U-^13^C]lactate by lactate dehydrogenase (LDH), an enzyme known to be highly active in tumor cells [14]. Since most [U-^13^C]lactate is released into the culture medium, its measurement over time by 1H-NMR allows an indirect evaluation of glycolytic activity.

In LS1034 cells, a tendency towards increased [U-^13^C]lactate formation was observed at longer incubation times. This result is in line with the [18F]FDG uptake data and supports the idea that LS1034 responded differently to butyrate when compared with the other two cell lines. Therefore, P-glycoprotein overexpression alone is unlikely to explain the distinct behavior of this model. In C2BBe1 and WiDr cells, the decrease in [U-^13^C]lactate release agreed with the uptake results. Taken together, these data suggest that, in WiDr and C2BBe1 cells, butyrate induced changes compatible with lower glycolytic activity, whereas LS1034 showed a divergent response and should be interpreted separately.

This conclusion is reinforced with the measurement of the ratio C3-Lac/C3-Ala. Higher values of this ratio indicate a more intense glycolytic pathway [29]. Exposure to butyrate decreased this ratio in the three cell lines, suggesting a reduction in glycolytic activity. However, in LS1034 cells, this decrease was not fully in line with the results obtained in the other assays, again indicating that the response of this cell line to butyrate may involve additional factors beyond a direct metabolic shift. Taken together, these data suggest that, in WiDr and C2BBe1 cells, butyrate induced changes compatible with the attenuation of glycolytic activity associated with the Warburg effect, whereas LS1034 showed a divergent response and therefore should not be interpreted in the same way.

**Figure 5:**
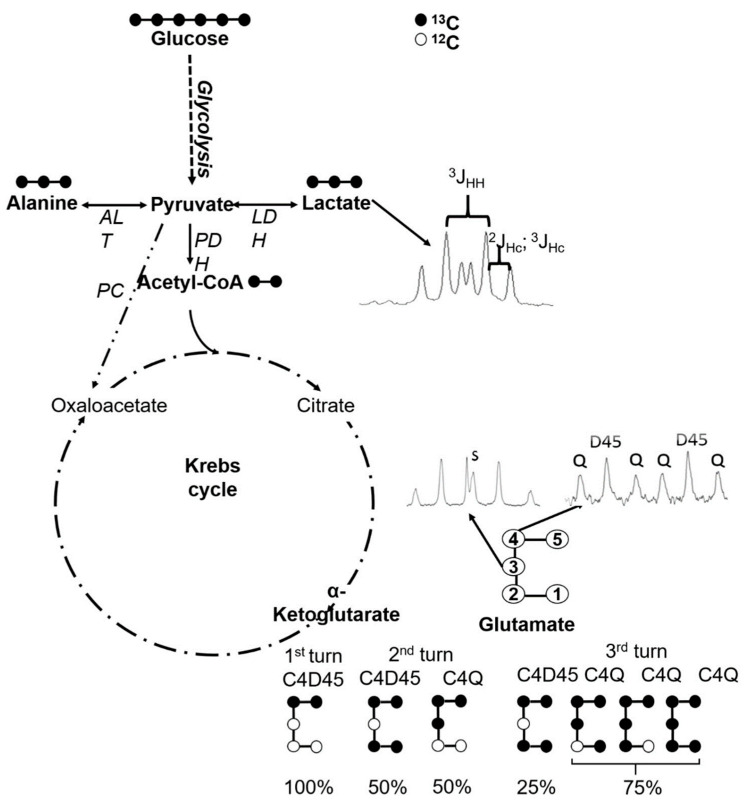
Use of [U-^13^C]glucose as a carbon tracer for monitoring glycolysis and Krebs cycle fluxes. After glycolysis, pyruvate may originate alanine, lactate, acetyl-CoA, or oxaloacetate. All these metabolic intermediates will have ^13^C incorporation. Numbers in the molecular structures represent the carbon position numbering. ALT (aminotransferase), LDH (lactate dehydrogenase), PDH (pyruvate dehydrogenase), and PC (pyruvate carboxylase). Q (quartet), D45 (doublet of glutamate carbon 4 corresponding to labeling at carbons 4 and 5), and S (singlet). Black circles represent ^13^C and white circles represent ^12^C.

The measurement of butyrate uptake showed that all three cell lines were able to uptake butyrate. Some studies suggest that the expression of some carriers associated with butyrate transport are diminished in colon tumors [7]. However, butyrate may go across cellular membrane by simple diffusion [30]. Therefore, the differences observed between cell lines do not seem to be related to an inability to uptake butyrate, but rather to the different concentrations used for each model. These concentrations were selected based on previously determined half maximal inhibitory concentration (IC_50_) values, reflecting the different sensitivity of the three cell lines to butyrate [19]. Accordingly, direct quantitative comparison between cell lines should be made with caution. In this context, the distinct behavior of LS1034 may be explained, at least in part, by a stronger cytotoxic effect under the experimental conditions used. Since LDH activity is widely used as a marker of cytotoxicity [31], the possibility that butyrate induced different degrees of cellular stress in the three models should be considered. Therefore, part of the differences observed between cell lines may reflect different degrees of cytotoxic stress in addition to metabolic reprogramming. This interpretation agrees with previous results from our group, showing that butyrate reduces viability and induces cell death-related changes in these colorectal cancer cell lines [19]. The ratio C3-Lac/C4-Glu measures the coupling between glycolysis and the Krebs cycle, being higher when less [U-^13^C]lactate reaches the mitochondria, where ^13^C is incorporated into glutamate through Krebs cycle turnover (Figure 5) [14]. In WiDr cells, the results suggest a better coupling between glycolysis and the Krebs cycle, consistent with a more oxidative profile. In the other two cell lines, no significant differences were observed.

Moreover, the ratio C4Q/C4D45 enables the evaluation of Krebs cycle turnover, since the C4 quartet only appears after at least two turns of the cycle with ^13^C incorporation (Figure 5) [14]. Thus, higher values of the C4Q/C4D45 ratio are associated with a higher number of complete cycles and, therefore, with higher oxidative metabolism. In the present study, butyrate promoted a decrease in the C4Q/C4D45 ratio in the three cell lines, which may be consistent with reduced Krebs cycle turnover under butyrate exposure. However, this interpretation should be made in the specific metabolic context of the experimental model. After entering the cell, butyrate is metabolized into two acetyl-CoA molecules by β-oxidation10, and Andriamihaja et al. (2009) showed that this may also occur in tumor cells [32]. Since butyrate was supplemented at natural-abundance ^13^C levels, the acetyl-CoA molecules derived from butyrate do not contribute to ^13^C enrichment, leading to dilution of the acetyl-CoA ^13^C enrichment pool and, consequently, to lower ^13^C incorporation into Krebs cycle intermediates and glutamate. Therefore, the decrease in C4Q/C4D45 should be interpreted together with the overall isotopomer pattern, since part of the observed effect may reflect dilution of ^13^C labeling in addition to reduced oxidative metabolism, and this parameter alone does not allow unequivocal conclusions regarding Krebs cycle turnover.

Blouin et al. (2011) proved that butyrate’s metabolization in tumor cells causes accumulation of lipid droplets and other macromolecules and is also associated with a reduction in cell proliferation [10]. This suggests that butyrate oxidation, together with its effects on glycolysis and other glucose-dependent metabolic pathways, may also interfere with biosynthetic pathways required for cell proliferation. In this context, the effects of butyrate on glycolysis, on the Krebs cycle, and on the coupling between these two pathways are consistent with an influence on cellular proliferation, since a more glycolytic phenotype is usually associated with higher proliferative activity. The anaplerotic stimuli, evidenced by the ratio C3-Glu/C4-Glu (Figure 4G), afforded by butyrate can be explained by the fact that butyrate dilutes the acetyl-CoA pool’s ^13^C enrichment, thus reducing overall C4-Glu enrichment, whilst C3-Glu still gets ^13^C enriched from the incorporation of [U-13C]pyruvate into the Krebs cycle through the anaplerotic pathway catalyzed by pyruvate carboxylase (PC). Also, butyrate is known to be a cellular differentiation promoter [33]. During differentiation, tumor cells may be using anaplerosis to develop lipid droplets and other macromolecules inherent to the increase in cellular complexity.

## 4. Materials and Methods

### 4.1. Cell Culture

Three colorectal cancer cell lines were acquired from ATCC, American Type Culture Collection (ATCC, Rockville, MD, USA): LS1034 (ATCC^®^ CRL 2158™, RRID:CVCL_1382), C2BBe1 [clone of Caco 2] (ATCC^®^ CRL 2102™, RRID:CVCL_1096), and WiDr (ATCC^®^ CCL 218™, RRID:CVCL_2760). All human cell lines have been authenticated using STR profiling within the last three years. Mycoplasma-free cells were used. Cell lines are from different colon localizations [14]. LS1034 was cultured in Roswell Park Memorial Institute-1640 medium (Sigma-Aldrich, Merck KGaA, St. Louis, MO, USA) supplemented with 1 mM sodium pyruvate. C2BBe1 and WiDr cell lines were cultured in Dulbecco’s Modified Eagle’s Medium (Sigma-Aldrich) supplemented with 0.25 mM sodium pyruvate. All cell lines were cultured in a medium with low glucose (5 mM) content supplemented with 10% heat-inactivated fetal bovine serum (Sigma-Aldrich) and 1% antibiotic/antimycotic (100 U/mL penicillin and 10 μg/mL streptomycin, Sigma-Aldrich).

To perform all the studies, the concentration of butyrate used was 6 mM for LS1034 cells, 15 mM for C2BBe1 cells and 3 mM for WiDr cells. These concentrations were selected based on previously established, cell-line-specific IC_50_ values obtained in the same colorectal cancer models after exposure to increasing butyrate concentrations [19]. This strategy aimed to apply biologically active doses adjusted to the intrinsic sensitivity of each cell line, while maintaining experimental conditions that were not expected to fully compromise cell viability. However, the use of cell-line-specific IC_50_ concentrations limits direct quantitative comparison between cell lines, since each model was exposed to a different butyrate concentration.

The selected timepoints were intended to capture different stages of the response to butyrate, including early functional effects on [^18^F]FDG uptake, intermediate changes in lactate release, and later adaptations in GLUT expression and intracellular metabolic labeling. Thus, the different assays were designed to provide complementary information on butyrate-induced metabolic changes rather than direct one-to-one mechanistic comparisons at identical timepoints. This approach was also supported by previous works in these colorectal cancer cell lines, showing dynamic lactate production over the first hours of incubation and distinct metabolic profiles between models [14].

### 4.2. [^18^F]FDG Uptake Studies

LS1034, C2BBe1 and WiDr cell lines were incubated in the presence and absence of butyrate, and uptake studies were performed following a previously described protocol [34]. After 1 and 4 h of butyrate incubation, 25 µCi/mL of [^18^F]FDG was added. At 5, 30, 60, 90, and 120 min, duplicate samples of 200 μL of cell suspension were collected to microtubes with iced phosphate buffer solution. Samples were centrifuged at 5600× *g* for 60 s, separating pellet from supernatant. Radioactivity of both fractions was measured in a well-type gamma counter (CAPINTEC CRC-15W, Capintec, Inc., Ramsey, NJ, USA) in counts per minute (CPM) and [^18^F]FDG uptake percentage was determined using Equation (1):

(1) $$\%\;Uptake=\frac{CPMpellet}{CPMpellet+CPMsupernatant\;}\times 100$$

### 4.3. Flow Cytometry

To evaluate the effects of butyrate on membrane and cytoplasmic expression of glucose transporters 1, 3, 5 and 12, flow cytometry was used. Cancer cells were incubated with butyrate for 1 and 24 h. Basal expression levels of transporters were also analyzed in untreated cells (no butyrate exposure).

The analysis was made as previously described. For membrane expression analysis, 1 × 10^6^ cells/condition were washed with PBS and incubated for 15 min at room temperature, protected from light, with anti-GLUT1-PE (R&D Systems, Minneapolis, MN, USA, FAB1418P), anti-GLUT3 (R&D Systems, MAB1415), anti-GLUT5 (R&D Systems, MAB1349), or anti-GLUT12 (Santa Cruz Biotechnology, Dallas, TX, USA, sc-161659). For GLUT3, GLUT5, and GLUT12, cells were subsequently washed and incubated for 20 min at room temperature, protected from light, with a PE-conjugated secondary antibody (Santa Cruz Biotechnology, sc-3738). For cytoplasmic expression analysis, 1 × 10^6^ cells/condition were fixed and permeabilized using Fixation and Permeabilization solutions from the IntraCell kit (ImmunoStep, Salamanca, Castile and León, Spain), according to the manufacturer’s instructions, and then incubated with the same primary antibodies and, when required, with the PE-conjugated secondary antibody, as described above.

Samples were acquired in a FACSCalibur flow cytometer (BD Biosciences, San Jose, CA, USA) and analyzed using Paint-a-Gate 3.02 software. Mean intensity of fluorescence (MIF) was determined in viable cells, selected based on forward- and side-scatter parameters (Supplementary Figure S1). Results are expressed as MIF, normalized to the corresponding untreated cells.

### 4.4. Nuclear Magnetic Resonance Analysis

For NMR analysis, cell lines were cultured in a medium purchased without glucose and supplemented with 5 mM of uniformly ^13^C-enriched glucose ([U-^13^C]glucose), with or without butyrate, towards monitoring possible metabolic effects of SCFA administration.

#### 4.4.1. Glycolytic Fluxes

Glycolytic fluxes were determined using a previously published procedure [14]. Acquisition of proton NMR spectra was performed on a 600 MHz Varian NMR spectrometer (Varian Medical Systems, Palo Alto, CA, USA) using a 3 mm indirect detection probe with a 3 s acquisition time, a radiofrequency pulse of 30°, and an interpulse delay of 10 s to ensure full relaxation of all nuclei in the sample. Unenriched lactate and [U-^13^C]lactate levels were measured for the times 0, 1, 3, 4, 6, and 8 h after glucose addition by deconvolution of its resonances using NUTSpro^TM^ (Acorn NMR Inc., Livermore, CA, USA). The distinction of [U-^13^C]lactate from unenriched lactate is possible due to direct ^1^*J*_HC_ and long range (^2^*J*_HC_ and ^3^*J*_HC_) heteronuclear scalar couplings. [U-^13^C]glucose is converted into two [U-^13^C]pyruvate molecules during glycolysis. After that, lactate dehydrogenase (LDH) converts pyruvate into [U-^13^C]lactate that will be released into the medium. Thus, determination of [U-^13^C]lactate levels is used as an indirect measure of glycolytic activity.

#### 4.4.2. Krebs Cycle Kinetics by ^13^C NMR Analysis of Cell Extracts

After 24 h of cell incubation in medium with [U-^13^C]glucose and in the presence or absence of butyrate, a methanol extract was prepared using an already described protocol [35,36]. Briefly, cells were washed with phosphate buffer solution, and then, 1.5 mL of a solution, MeOH/H_2_O 80:20 (*v*/*v*), was added. The metabolites were dissolved and separated from cellular waste by centrifugation, for 5 min at 5725 G. The MeOH:H_2_O extract was dried and redissolved in 160 μL of D_2_O (99.9%) plus 40 μL of a sodium fumarate solution (10mM) in phosphate buffer (100 mM), used as an internal standard, and ^13^C-NMR spectra was acquired. Typical acquisition parameters included a radiofrequency pulse of 45°, an acquisition time of 1.5 s and an interpulse delay of 3 s to ensure full relaxation of all aliphatic carbons in the sample. ^13^C-NMR spectra allow a dynamic vision of cell metabolism by analyzing the ^13^C incorporation on intracellular metabolic intermediates. According to Figure 5, glutamate ^13^C incorporation correlates with Krebs cycle activity, and the measurement of the appearance of ^13^C isotopomers of intracellular glutamate enables the evaluation of possible butyrate effects on Krebs cycle, anaplerotic, and pyruvate cycling fluxes [14,37].

To measure the coupling between Krebs cycle and glycolysis, a ratio between lactate and glutamate ^13^C incorporation was determined (C3-Lac/C4-Glu). To evaluate the effect of butyrate on the redox state of cytosol, a ratio between intracellular lactate and alanine levels (Lac/Ala) was determined. The use of these ratios to derive relevant metabolic information was already validated and described in other publications [14,37].

### 4.5. Statistical Analysis

Statistical analysis was performed using IBM SPSS software v.24.0 (IBM Corporation, Armonk, NY, USA). The descriptive analysis of quantitative variables under study was performed by calculating estimators of central tendency, dispersion, and location. In the inferential analysis, normal distribution of quantitative variables was evaluated according to Shapiro–Wilk test.

For comparisons between two independent samples, Student’s t-test was used in the case of normal distribution of values, or Mann–Whitney test otherwise. Comparisons between more than two groups were performed using one-factor test (ANOVA) or Kruskal–Wallis test (according to criteria previously mentioned). Multiple comparisons were performed according to Bonferroni correction.

To analyze uptake curves, MATLAB R2014a program was used. The values obtained were fitted to an exponential model:

(2) $$U\left(t\right)=A\cdot \left(1-e^{\ln\left(2\right)\cdot t/T_{50\%}}\right)$$

where U(t) is the uptake at t time (minutes) and A is the maximum uptake corresponding to steady state. This model allowed the determination of the time required to reach the uptake corresponding to A/2, named here as T50%.

The experimental values obtained for lactate production using NMR analysis were adjusted to a linear model using the software OriginPro v. 8.0 (OriginLab Corporation, Northampton, MA, USA):

(3) y=m·x

where m represents the straight slope. This parameter was compared between control condition and treatment according to Student’s t-test for independent samples.

A significance level of 5% was adopted for all comparisons.

## 5. Conclusions

Our results suggest that butyrate can interfere with metabolic alterations associated with the Warburg effect in WiDr and C2BBe1 cells, suggesting a less glycolytic profile in these models. This interpretation is summarized in the graphical abstract where, in the presence of butyrate, some metabolic alterations associated with the Warburg effect are attenuated, namely glucose uptake, glycolytic activity, and changes in Krebs cycle turnover.

In LS1034 cells, butyrate induced increased [^18^F]FDG uptake and a tendency towards increased lactate formation at longer incubation times, contrasting with the response observed in WiDr and C2BBe1 cells. This behavior may reflect the distinct metabolic profile of LS1034, together with a stronger cytotoxic effect of butyrate under the experimental conditions used. Therefore, LS1034 should be considered a distinct model in the interpretation of the metabolic effects of butyrate. The interference of butyrate with glucose oxidation has been a target for research with promising results [10], as well as its interference with some GLUTs isoforms [38]. Similar effects have also been described for other HDAC inhibitors [39]. Butyrate, as well as other HDAC inhibitors, has also been described as a differentiation promoter. In this context, the implication of differentiation in the metabolic changes observed here cannot be excluded. Since cancer stem cells are associated with a more exacerbated glycolytic profile [40,41], the promotion of differentiation and the reduction of the hyperproliferative phenotype induced by butyrate may increase susceptibility to other therapies [19,42].

These findings support further research on the biological role of butyrate and other soluble dietary fiber-derived metabolites in colorectal cancer. Since butyrate is generated in the colon by microbial fermentation of soluble dietary fiber [11], future studies should investigate whether fiber-rich dietary patterns may contribute to colorectal cancer prevention or to modulation of tumor metabolism. In this context, foods such as whole grains, legumes, fruits, and vegetables may be of particular interest as dietary sources associated with increased fiber intake.

## Figures and Tables

**Table 1:** Average values of the parameters A (% of maximum uptake, steady state) and T (time required to reach 50% of [^18^F]FDG uptake, is equal to A/2, in minutes) for the three cell lines studied under different incubation times with butyrate (0, 1, or 4 h). A and T values are represented as mean (confidence interval).

Cell Line	Time (h)	A (Uptake %)	T (minutes)	R^2^
LS1034	0	1.83 (1.68; 1.98)	23.40 (17.30; 29.50)	0.97
	1	2.90 (2.72; 3.08)	36.02 (30.76; 41.28)	0.99
	4	2.54 (2.32; 2.75)	18.58 (12.84; 24.32)	0.95
C2BBe1	0	7.22 (6.23; 8.20)	55.54 (41.85; 69.22)	0.99
	1	3.71 (2.49; 4.93)	37.73 (9.52; 65.94)	0.83
	4	2.66 (2.24; 3.09)	16.61 (5.96; 27.26)	0.8
WiDr	0	11.82 (6.82; 16.82)	92.02 (35.72; 148.33)	0.96
	1	4.11 (3.82; 4.41)	47.10 (40.39; 53.81)	0.99
	4	2.95 (2.61; 3.29)	37.16 (27.29; 47.03)	0.98

## Data Availability

The original contributions presented in this study are included in the article and Supplementary Materials. Further inquiries can be directed to the corresponding author.

## Supplementary Materials

The following supporting information can be downloaded at: https://www.mdpi.com/article/10.3390/ijms27083696/s1.

## Abbreviations

The following abbreviations are used in this manuscript:

C3	Carbon 3
C4	Carbon 4
^18^F	Fluorine-18
[^18^F]FDG	Fluorine-18 fluorodeoxyglucose
GLUT	Glucose transporters
HDAC	Histone deacetylase
LDH	Lactate dehydrogenase
MIF	Mean intensity fluorescence
NMR	Nuclear magnetic resonance
SCFA	Short-chain fatty acid
