# Supplementary figures and images for: Preventive Effect of Butyrate in Colon Cancer Cell Metabolism
Source: Int J Mol Sci. 2026 Apr 21;27(8):3696. doi: 10.3390/ijms27083696 (PMC13116300; doi:10.3390/ijms27083696)

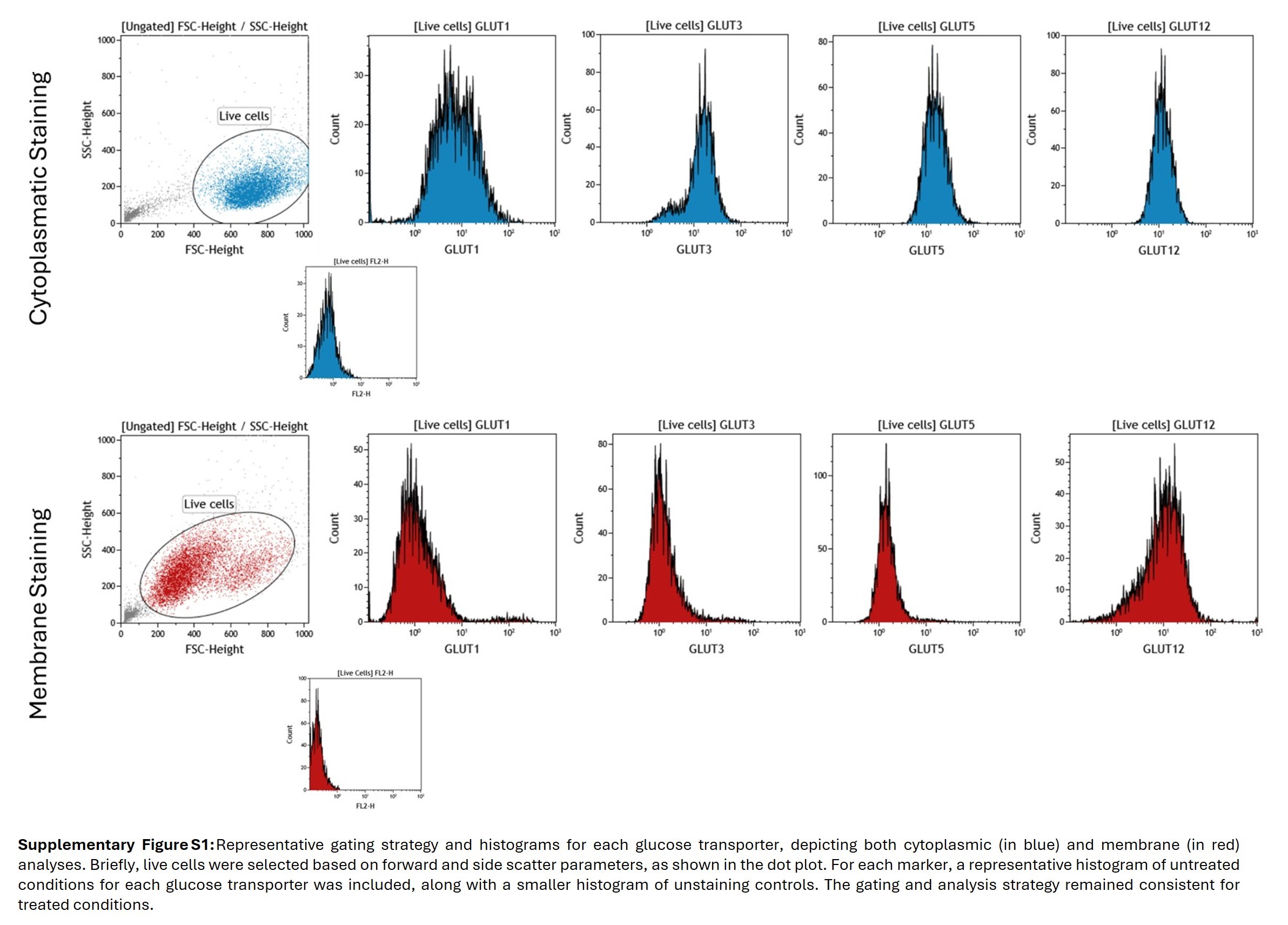

Supplement: Supplementary file 1 [file ijms-27-03696-s001.zip › ijms-4150668-supplementary.jpg]
